# The Benefits and Challenges of Antibiotics–Non-Steroidal Anti-Inflammatory Drugs Non-Covalent Reaction

**DOI:** 10.3390/molecules28093672

**Published:** 2023-04-23

**Authors:** Ilma Nugrahani, Diar Herawati, Marlia Singgih Wibowo

**Affiliations:** School of Pharmacy, Bandung Institute of Technology, Bandung 40132, Indonesia

**Keywords:** antibiotics, fluoroquinolones, multi-component solids, non-covalent reaction, NSAIDs, physicochemical properties, potency, salt, solubility, toxicity, stability

## Abstract

Recently, non-covalent reactions have emerged as approaches to improve the physicochemical properties of active pharmaceutical ingredients (API), including antibiotics and non-steroidal anti-inflammatory drugs (NSAIDs). This review aimed to present and discuss the non-covalent reaction products of antibiotics, including salt and neutral multi-component solid forms, by framing their substituents and molar ratios, manufacturing techniques, characterization methods, benefits, potency changes, and toxicity, and is completed with an analysis of the development of computational models used in this field. Based on the data, NSAIDs are the most-developed drugs in multi-component system preparations, followed by antibiotics, i.e., antituberculosis and fluoroquinolones. They have reacted with inorganic elements, excipients, nutraceuticals, natural products, and other drugs. However, in terms of treatments for common infections, fluoroquinolones are more frequently used. Generally, NSAIDs are acquired on an over-the-counter basis, causing inappropriate medication. In addition, the pKa differences between the two groups of medicine offer the potential for them to react non-covalently. Hence, this review highlights fluoroquinolone–NSAID multi-component solid systems, which offer some benefits. These systems can increase patient compliance and promote the appropriate monitoring of drug usage; the dual drug multi-component solids have been proven to improve the physicochemical properties of one or both components, especially in terms of solubility and stability. In addition, some reports show an enhancement of the antibiotic activity of the products. However, it is important to consider the possibility of activity changes, interaction, and toxicity when using drug combinations. Hence, these aspects also are discussed in this review. Finally, we present computational modeling, which has been utilized broadly to support multi-component system designs, including coformer screening, preparation methods, and structural modeling, as well as to predict physicochemical properties, potency, and toxicity. This integrated review is expected to be useful for further antibiotic–NSAID multi-component system development.

## 1. Introduction

Antibiotics have been some of the most used drugs in the last decades. Consequently, microorganism resistance has occurred due to their longtime usage. In addition, antibiotics must target microbes, but should be safe for human cells; hence, their toxicity must also be considered. On the other hand, low solubility and instability are typical problems for some antibiotics, which may cause the drug’s content not to reach the appropriate levels. Next, chemical degradation also may produce toxicity and allergens. Thereafter, efforts have been made to find more selective, potent, safe, and effective antibiotics. However, the complexity of finding new drugs, from modeling and synthesis to clinical testing and formulation, makes the process slow [1].

The development of new drugs requires reagents, i.e., starting chemicals, catalysis, solvent organics, etc. In addition, the synthesis of new drugs requires optimization to obtain a high percentage of yield or purity. Besides, the process may create chemical waste and side products. After its synthesis, a new synthetic drug should be evaluated by more extended pharmacological testing, including toxicity, mutagenicity, clinical testing Phase I, II, and III, etc., before it can be accepted as a suitable active pharmaceutical ingredient. These, often long, steps of drug discovery are costly because they consume a significant amount of resources and time.

In addition to traditional drug development strategies, combining existing drugs with other compounds to create multi-component solids is becoming an increasingly interesting approach to improving drug performance. This technique requires less solvent and is easier to perform than other methods, making it an attractive option. The process involves low-energy, non-covalent reactions that don’t affect the drug–receptor binding, making it ideal for use in the body. By using established drugs to create multi-component systems, certain clinical tests may be avoided, making the solid engineering technique even more desirable. In addition, no reagents are used, high yields are obtained, and almost no waste or residue is yielded. However, it’s important to carefully consider the interaction cases when combining APIs in a multi-component solid.

Computational structure–activity relationship modeling has been progressively developed to predict antibiotics’ structure, potency, resistance, mechanism of action, and toxicity [2,3]. One study proved that similar structures tend to show similar physicochemical properties. For example, all fluoroquinolones may have photosensitivity, hygroscopicity, and pH-dependent solubility, confirming the experimental data [4,5]. Hence, many efforts have been performed to overcome those lack of properties. One of the common strategies is reacting antibiotics with inorganic chemicals (metals/halogens) or organic compounds, such as other drugs, excipients, nutraceuticals, etc. Moreover, in addition to improving the solid characteristics, experiments have shown that fluoroquinolone antibiotics can enhance potency by changing the polarity and penetrability of the bacteria’s membrane through non-covalent reactions [6,7]. 

On the other hand, aside from antibiotics, infected patients usually consume anti-inflammatory agents to overcome inflammation symptoms. NSAIDs (non-steroidal anti-inflammatory drugs), which have relatively few adverse effects, have become first-choice drugs, and are available widely in various dosage forms. However, they generally have low solubility. Furthermore, some are physically unstable, i.e., they show polymorphism and pseudopolymorphism, which change their solid characters. In addition, they may also be chemically unstable. NSAIDs also have exhibited non-covalency with various counterparts in the multi-component solids, which could explain their lack of physicochemical properties. Furthermore, combining both medicines to enhance patient convenience and compliance and improve their physicochemical properties without changing their activity is an exciting prospect. This effort is supported by the fact that many NSAIDs tend to be acidic [8]; inversely, many antibacterial compounds are basic [6,7], or they may be amphoteric [6,7,8]. Hereafter, many antibiotics–NSAID combinations are feasible, resulting in non-covalent interactions and the production of multi-component solid systems. 

The first and crucial step in creating multi-component structures is determining the coformer or stoichiometric ratio. Once this is established, appropriate methods can be used to react with the components, including slow evaporation, fast evaporation, neat grinding, liquid-assisted grinding, slurry, and co-precipitation [9,10,11]. Additionally, greener methods are currently available, such as hot melt extrusion (HME) [12], supercritical carbon dioxide [13], sonication slurry technique [14], sublimation [15], and microwaving [16,17]. After this process, reliable analysis methods can be used to confirm the new solid phase formation. Firstly, the thermal analysis may be performed using an electrothermal measurement [6,7], thermogravimetry analysis (TG) [18,19], differential thermal analysis (DTA) [19,20], and differential scanning calorimetry (DSC) [6,7,11,12,21] can be used to identify a new phase. An endothermic curve and the melting point may represent the water released from the hydrate system. Meanwhile, recrystallization is shown by an exothermic curve and degradation, with irregular exothermic curves [6,7,20]. Therefore, a new structure’s thermogram has a different pattern from a single component or physical mixture. Thermal analysis has long been used in pharmaceutical research to identify and detect purity based on phase thermodynamic principles [21,22,23].

However, only powder X-ray diffraction (PXRD) can precisely identify the new solid phase formation based on the diffractogram pattern. The specific distance and intensity represent the unique electron position of the atoms in the new crystal lattice. Hence, different diffractogram patterns from the starting materials indicate the multi-component crystal structure formation. Next, a solid analysis should be conducted to fix the structure and kind of interaction. Various methods can be used, including vibrational spectroscopy techniques such as Fourier transform infrared spectroscopy (FTIR), Raman spectroscopy, and Terahertz spectroscopy, as well as nuclear magnetic resonance (NMR) [24,25].

This review aims to present and discuss various antibiotic multi-component structures and their preparation methods, challenges, and benefits. First, we highlight antituberculosis agents as the first antibiotic multi-component systems. Then, we discuss fluoroquinolone multi-component solids, which are the currently most-reported structures. However, these drugs can be poorly soluble and unstable in light and humid conditions. It has been found that N-methyl-amine-piperazine is responsible for photodegradation and requires protection [26,27,28,29]. On the other side, NSAIDs are insoluble, and some are unstable, such as indomethacin and diclofenac acid [8]. Hence, in another section, we describe combinations with NSAIDs, since they are commonly used to treat infected patients [30,31,32], completing the paper with considerations regarding the activity and toxicity caused by drug–drug interactions [31,32,33]. 

Finally, the in-silico method has been recognized. It is currently preferred, as a green, efficient, and effective tool for designing new multi-component solids and calculating their physicochemical properties [34,35,36,37,38], as well as for predicting their activity and toxicity [39,40]. This review attempted to provide comprehensive information and is expected to encourage good design and experimental research on developing antibiotic–anti-inflammatory combinations. 

## 2. Antibiotics

Based on their chemical structure and mechanism of action, antibiotics are divided into six groups: (1) cell wall synthesis inhibitors, including the beta-lactam class (e.g., penicillin, cephalosporin, and carbapenem) and dissimilar agents, such as cycloserine, vancomycin, and bacitracin [41]; (2) those that directly work on the cell membrane of the microorganisms, to increase permeability and lead to leakage of intracellular compounds, which include detergents such as polymyxin, and polyene antifungal agents, which bind to cell-wall sterols (e.g., nystatin and amphotericin B) [42]; (3) 30S or 50S ribosomal subunit disruptors, which reversibly inhibit protein synthesis, and commonly work bacteriostatic agents (e.g., chloramphenicol, the tetracyclines, erythromycin, clindamycin, streptogramins, and linezolid) [43]; (4) agents that bind to the 30S ribosomal subunit and alter protein synthesis, which are generally bactericidal (e.g., the aminoglycosides) [44]; (5) agents that affect bacterial nucleic acid metabolism, such as rifamycin, rifampin and rifabutin, and inhibit RNA polymerase [45,46]; the quinolones, which inhibit topoisomerases [47]; (6) antimetabolites, including trimethoprim and the sulfonamides, block essential enzymes of folate metabolism [48]. 

Meanwhile, based on indications, antibiotics are divided into sulfonamides, trimethoprim, sulfamethoxazole, quinolones (nalidixic acid, ciprofloxacin, delafloxacin, gatifloxacin, levofloxacin, moxifloxacin, norfloxacin, ofloxacin, pefloxacin, sitafloxacin), penicillin, cephalosporins, aminoglycosides, macrolides, clindamycin, quinupristin/dalfopristin, linezolid, vancomycin, teicoplanin, daptomycin, bacitracin, polymyxin, and mupirocin [1,48]. Those antibiotic classes are listed in Table 1. Many new-generation antibiotics are still being developed.

## 3. Antibiotic Multi-Component Structure Development

A multi-component crystal is a solid phase consisting of more than one kind of molecule stoichiometrically. This solid phase comprises intermolecular bonds between the components, which are also influenced by their intramolecular chemical bonding. Based on the type of interactions, the multi-component solids are divided into three groups: salts, cocrystals, and hydrate/solvate. The APIs can be an acid, base, or neutral compound, and can combine with another drug, nutraceutical, excipient, or substituent to form a multi-component crystal. Salt or cocrystal formation depends on the “rule of pKa”, which states that components with a pKa value of less than three will produce cocrystals; salt requires a pKa value higher than three. In addition, salt cocrystals can also be produced as a result of the reaction between neutral multi-component systems, acids or Schiff bases, and metal atoms [50]. 

Multi-component solids may be built by combining a non-soluble component with a water-soluble substituent via hydrogen bonding to attract the water molecules, increasing the solubility. The solid-state arrangement may improve a wide range of APIs’ physicochemical properties, including their solubility, stability, flowability, and permeability. Many also enhance activities and performances, as recently reported [30,51,52,53,54,55]. However, the first benefit is solubility enhancement, which is stated to be the most crucial characteristic of the particles. The solubility increase depends on the starting material’s properties, interaction type, and crystal structure conformation. For example, pymetrozine multi-component systems showed a four-fold improvement in these parameters [54], and nicorandil multi-component crystals showed a 1.9–5.2-fold improvement [55]. Meanwhile, the solubility of NSAIDs after salt formation with fluoroquinolones reaches values tens to hundreds of times higher than their parent compound [30]. 

Both ionic and non-ionic reactions of the fluoroquinolone group, i.e., ciprofloxacin and levofloxacin, with some substituents increase the solubility of these compounds [6,9], as well as showing increased stability towards humidity and light, which is the main advantage of these systems. For example, the combination of levofloxacin–citric acid [6] and levofloxacin–dihydroxybenzoic acid [7], and ciprofloxacin with cyclodextrin and cucurbit (7)-uril (CB7) improves photostability up to three-fold [27]. 

### 3.1. Salt/Ionic Multi-Component Structure

Salt is formed by the different ionic/charge compounds, which transfer hydrogen atoms between acid and base moieties, and may consist of inorganic and organic counterparts to the host molecule. This structural derivation has been applied for a long time, significantly increasing drug solubility and stability, physically and chemically. In addition, the changes observed in antibiotics’ physicochemical properties after salt formation can improve in terms of antibacterial potency, as shown by levofloxacin in combination with antioxidants, citric acid [6], and dihydroxybenzoic acids [7], as well as by ciprofloxacin in combination with NSAIDs [30,31]. 

### 3.2. Neutral/Non-Ionic Multi-Component Structure or Cocrystal

Although it was discovered in 1844 and characterized in 1958, the term ‘cocrystal’ was first used in 1963 by Lawton and Lopez. This term refers to a combined system of solid compounds in a crystal lattice, formed due to non-ionic intermolecular bonding with at least a neutral organic counterpart, referred to as a “co-former”. A coformer, particularly non-API, should be pharmacologically neutral, non-toxic, and considered a GRAS (Generally Recognized as Safe) compound. The API and the paired combination must have different polarities to form cocrystals, which then interact in the binding site, known as a “synthon”. Supramolecular synthons may be a homo-synthon (same functional group) or hetero-synthons (various moieties). Drug–drug, drug–nutrient, drug–vitamin, and drug–excipient combinations can cause the formation of a neutral multi-component structure. The cocrystals may involve hydrogen or halogen bonding in two or more different neutral compounds. Furthermore, combining an ionic component with a cocrystal can form an “ionic-cocrystal” with a charge [18,50]. 

However, antibiotics may also form neutral and ionic multi-component solids, i.e., ciprofloxacin with dicarboxylic acids [9,21] and salicylic acid [31].

### 3.3. Hydrate and Solvate

Another type of multi-component solid is formed when a molecule compound interacts with an organic solvent or water molecule and builds a stoichiometrically neutral structure, producing a solvate and hydrate, respectively. Solvate and hydrate variations depend on concentration, temperature, humidity, and pressure. Naturally, many antibiotics have hydrate forms, which are commonly more stable than solvates, with different physicochemical properties, such as density, the molecular weight of the crystal, solubility, stability, hygroscopicity, etc. [18,26,56]. More importantly, the solvent or water portion should adjust the dose required, which is often unfixed or unpredictable. 

Fluoroquinolones have many sites with the potential to form hydrogen bonds and, often, more than one lattice hydrate forms. For example, levofloxacin and ciprofloxacin may be observed in hemihydrate and monohydrate phases [6,7,19,26,56]. Technically, mainly pseudo-polymorphs are obtained through recrystallization using a solvent. However, as an important note, the degree of conformational flexibility of the molecule is very high, which affects the screening process, and the results of hydrate arrangement are unpredictable [56,57,58,59]. Therefore, an integrative analysis should be performed to determine the true pseudo-polymorphs, including PXRD, thermal analysis, i.e., DSC elaborated with thermogravimetry, and, finally, SCXRD. 

### 3.4. Multi-Component System Preparation Methods

Several methods can be used to create a new solid structure, which can be categorized into three techniques: dissolving, grinding, and heating. The dissolution method commonly produces single crystals and comprises slow evaporation, rapid evaporation, cooling, chemical reactions, spraying, and freeze-drying [8,9]. In this method, active pharmaceutical ingredients (APIs) and a coformer are mixed and entirely dissolved in the solvent, then evaporated to produce salts or neutral combination compounds. Under the solution conditions, the components are mixed accurately, with the solvent facilitating the interaction between them. Next, the solvent evaporates when the solution is supersaturated, and hydrogen bonds or ionic interactions are formed between the molecules. This process goes slowly or quickly, depending on the contact area between the solvent and the atmosphere. In addition, the evaporation process may be accelerated by increasing the temperature and pressure, for example, by storing the solution in an evaporator chamber or using a rotavapor with the appropriate temperature setting. In terms of kinetic energy, the size of the multi-component crystal will be bigger if evaporation runs slowly. Conversely, the multi-component crystal will be smaller if the oration occurs quickly. The evaporation method produces thermodynamically stable crystals but requires a large amount of solvent. 

Another evaporation method is a cooling technique at a low temperature to accelerate the saturated condition and produce more crystals spontaneously. Subsequently, the freeze-drying methods involve dispersing an unsaturated solvent into a nozzle using nitrogen gas. These processes aim to remove the unsaturated solution of drugs and co-formers through extreme conditions, such as the use of high pressure and low temperature, to cause the other parts become very saturated; as such, the evaporation process runs faster [8,9]. 

Next, grinding methods are divided into neat and solvent-assisted grinding. First, neat grinding is done in a mortar to mix and press the component thoroughly, without any solvent. This method produces large numbers of multi-component systems and is quicker than evaporation. However, the homogeneity of this method could be better in large-scale production. The second method is liquid-assisted grinding, using a small amount of solvent in the milling process. The solvent acts as a catalyst without waste production [60]. Other researchers report that a combination of ciprofloxacin–isonicotinic acid can be built by mechano-chemical synthesis, in order to improve stability [61]. This mechanochemical technique is recommended, as it is environmentally friendlier than evaporation due to the reduced usage of organic solvents. However, large-scale production homogeneity is still a challenge.

Heating methods include hot melt extrusion (HME) [12], microwave methods [16,17], and isothermal slurry [1]. High energy activates electrons and accelerates the reaction. Hot melt extrusion uses heat and pressure to melt the mixture in the extruder. The interactions between the drug and the excipient occur as they melt. This method is faster and reduces organic solvent usage. It has been informed that this technique was also tested on ciprofloxacin–isonicotinic acid, as well as the neat grinding method [61]. HME is fast but requires thermo-stable compounds, similar to microwave methods. 

Next is the microwave method, which differs from HME in its energy source. HME uses a “stove” apparatus, heat conductance, and pressure; a microwave uses the radio-electromagnetic wave, which vibrates and excites the atoms and molecules. Microwaving accelerates this process and maximizes the multi-component solid yield significantly. For example, the diclofenac-L-proline cocrystal can be produced using a microwave in 30 s [16]. Last, the slurry method is used to suspend the components using water or other solvents, and then heat them, in order to intensify the reaction in wet conditions [11,12]. The powder or crystal yields that can be separated from the solvent make this technique greener than evaporation. This method has been used to synthesize caffeine/maleic acid co-crystals, supported by ultrasonication [12]. This method is restricted only to the undegradable compounds in the solution under hot conditions. 

Besides conventional methods, some developed techniques exist, such as the supercritical process [13], sublimation [15], etc. The supercritical method uses a liquid gas, such as carbon dioxide, facilitating intensive contact between the reacting components. Supercritical carbon dioxide acts as a co-solvent and antisolvent, replacing conventional co-crystallization by selecting greener solvents, and may produce fine and uniform particles. The gas antisolvent is the most suitable in the supercritical fluid (SCF) co-crystallization process, possibly due to its similarities to conventional antisolvent methods. It was reported that solvent selection was one of the most critical parameters in producing the appropriate yield, phase purity, and polymorphs, to different extents. In addition, a systematic solubility study was required to optimize the processes [13]. 

Meanwhile, sublimation is a process that facilitates the formation of multi-component systems by providing the necessary energy to transform a mixture of solid components into a gas and subsequently condensing it into a multi-component solid form. Commonly, an oven is used simply in the hot melt extrusion and sublimation methods. However, this technique has been used to successfully isolate 4-fluorophenol–p-benzoquinone cocrystals, with different polymorphs than those in the conventional method’s yield, showing that sublimation determines the phase space of cocrystals [14].

### 3.5. Multi-Component Systems Analysis and Characterization Methods

The standard instruments and techniques used to confirm the new solid phase formation are microscopes (binocular/polarized/electron microscope, etc.), thermal analysis (electrothermal, DSC, TGA), vibrational spectrophotometry (FTIR, Raman, Terahertz spectroscopy), NMR, and X-ray diffractometry (PXRD AND SCXRD). Thermal analysis observes the thermal properties of solid compounds. Next, structural analysis methods, such as vibrational spectroscopy (FTIR, Raman, and Terahertz) and NMR, analyze structural changes that occur after a successful reaction. Finally, SCXRD determines the 3D conformation to show the atoms/molecules’ position, distance, and angle position of atoms and molecules. 

In this subsequent, we present the solid instrumentations used to characterize the new multi-component phases.

#### 3.5.1. Conventional and Semimanual Thermal Analysis

Every material has a unique energy that is reflected in its physicochemical properties. Thermal analysis, which observes the phase transitions of a substance under heating, such as melting point, recrystallization, oxidation, and degradation, is one of the oldest methods used for material characterization. In the past, conventional apparatuses such as a melting block, Thiele apparatus, or bomb calorimeter have been used to observe those parameters. 

A simple thermal method in solid-state observation is thermal microscopy, published by Koffler many years ago [62], but it is still relevant today. An electrothermal microscope is a compact device used to observe the thermal profile of a solid phase, which is heated in order for one to observe phase changes or the transition point, including wetting due to the water release from the hydrate, recrystallization, and fusion, as well as determines a solid material’s melting point, degradation, and a range of other physical and chemical changes that occur during the heating. This method uses electrical energy as a heat source and a heat conductor. The sample only needs to fill a one-sided capillary tube. The starting temperature and the heating rate are set digitally. The components of the electrothermal device are the sample hole, capillary tube, heater, a magnifying lens for observation, and an integrated lamp. A visual hole allows the researcher to observe the solid-state changes. Multi-component systems show a different thermal profile than the single component and may have a lower [18,28] or higher melting point. This method is environmentally friendly as it requires no solvents, is low cost and easy to perform [6,7]. 

#### 3.5.2. Modern Calorimetry: DSC and DTA/TG

A DSC is a calorimeter that measures the heat flow rate versus the temperature increase. This method is used to observe the thermal characteristics of a solid material in detail, such as the glass transition, water/solvent release from a pseudopolymorphism, fusion/melting point, recrystallization at high temperature, and decomposition, as well as to observe subtle physical changes, such as the glass transition temperature and eutectic point [22,23,63,64]. The DTA measures the temperature difference that occurs during heating and is commonly combined with a TG in one device. Finally, the TG measures the mass decrease resulting from heating and is the primary method used to characterize solvates/hydrates, supporting DSC and DTA data [6,7,18,65]. 

DSC and DTA thermograms will show endothermic and exothermic curves, representing the energy change during the phase transformation. For example, the glass transition is demonstrated by an endothermic blunt curve, a broad endothermic peak indicates water or solvent molecules release, the melting point shows a sharp endothermic curve, a sharp exothermic curve indicates recrystallization, and oxidation/degradation is indicated by some irregular curves [20,21,22,23]. Currently, almost all the multi-component development reports show the DSC data, indicating that this method is adequate for identifying a new entity with a specific energy. 

Depending on their lattice thermodynamics, a multi-component crystal may have a lower or higher melting point/degradation temperature than the starting compounds. One can observe the energetical changes resulting from the interaction energy present in the new multi-component solid phase. For instance, the formation of the levofloxacin–phthalimide cocrystal and levofloxacin–caffeic acid salt led to a reduction in melting point, with values of 172–176 °C and 150–152 °C, respectively, compared to levofloxacin and the coformers, which all have melting points above 200 °C [53]. This indicates significant interaction of energy between the components in the new solid phase. On the other hand, levofloxacin salts, as yielded from reactions with citric acid [6] and dihydroxybenzoic acid [7], have higher melting points than those of the starting materials. In addition, levofloxacin salts are thermolabile and exhibit a degradation profile before melting, which is similar to the single antibiotic but at different temperatures [6,7,21,28].

#### 3.5.3. Vibrational Spectroscopy Analysis

FTIR is commonly used in the characterization and identification of solid states. This method can recognize functional groups involving various bonds, including ionic, covalent, van der Waals, and hydrogen bonds. The working principle of this instrument is to measure the interaction of infrared waves with the bond energy between atoms. The transition of intramolecular vibrational energies (vibrations in the bonds between atoms) will be measured in terms of the amount of radiation contained in the infrared waves emitted by the source. Molecular vibrations are classified into two main groups: stretching and bending. The frequency of infrared waves is expressed in units of wave number (cm^−^^1^); the infrared wave spectrum has a wave number region of 14,000 cm^−^^1^ to 10 cm^−^^1^, but the region used for compound analysis is only the middle region, with a wavelength of 2.5–50 µm or wave number 400–4000 cm^−^^1^. 

The vibrations in each molecule are unique, so this method is beneficial for identifying a compound. The specific spectrum of each type of bond is called fingerprint vibrations [8]. For example, in the case of hydrate forms, the presence of water molecules can be identified by characteristic peaks in the infrared spectrum, such as the hydrogen bonding stretching vibrations in the range of 2500–3500 cm^−1^. Next, the interaction between –COO and –NH synthons in a multi-component system is represented at 1500–1900 cm^−^^1^, as well as in the fingerprint area of 400–1500 cm^−^^1^ [8,60,64,65,66,67]. Hence, FTIR detects multi-component system formation by showing a distinctive spectrum. The salt reaction commonly exhibits a more apparent spectrum [7,9] than cocrystals do [18], reflecting their higher interaction energy [9]. This method is sensitive to moisture, but water molecules in a hydrate lattice have a specific band, which is narrower than the broad spectrum of the entrapped water on the particle’s surface. 

Secondly, Raman spectroscopy is a useful tool for analyzing changes in the solid structure, such as hydrate formation, by identifying specific peaks. Raman spectroscopy has also been utilized to detect the presence of water molecules in the crystal lattice [64], for example, by analyzing the unique ciprofloxacin salicylate 1.75 hydrate [65]. Finally, Terahertz spectroscopy also has been used to identify new solid phase construction; for example, it has been previously used to determine pyrazinamide–3-hydroxybenzoic acid [68].

#### 3.5.4. NMR

NMR is an adequate method for analyzing chemical structures two-dimensionally and has been available for measuring liquid and solid samples (solid-state NMR/SSNMR). NMR spectroscopy interacts with the nuclei of atoms with radio electromagnetic (REM) waves, then profiles the resonance frequency yielded. Solid-state NMR may identify and quantify the multi-component solids, of both a crystalline and amorphous nature, by using an irradiation frequency of 500–1000 MHz, with the C and H positions and their bindings represented by the chemical shift in the spectra. Hence, SSNMR deals with solid-structure elucidation, amorphous and crystal characterization, dynamics, and stability. The structure can be solved accurately by reading the exact position of the resonance of the distinct nuclei. NMR has been used in solid-state development to ensure the formation of new interactions. For example, the reaction of levofloxacin with citric acid [6] and dihydroxybenzoic acid [7] was identified by showing distinctive spectra compared to the starting materials. This method is beneficial in determining the structure in the case where an appropriate single crystal cannot be isolated, and when FTIR and SCXRD cannot predict the structure interaction.

#### 3.5.5. X-ray Diffractometry 

The X-ray diffractometry observes the change of atomic or molecular order and position, represented by their distance and angle in the lattice structure. X-ray diffraction is an analytical method based on the ability of a crystal to diffract X-rays and produce a characteristic pattern, which allows for an in-depth study of the solid phase structure. This diffraction method utilizes the constructive interference event of monochromatic X-rays. When X-ray radiation reaches atoms in the crystal structure, the high energy of the radiation will cause electrons to move to a higher level. However, this transfer of electrons does not last long because the state is unstable, so the electrons will return to their ground state and release light with identical wavelengths. This phenomenon is known as Rayleigh scattering. 

X-ray diffractometry is specific to analyzing crystalline samples because the amorphous forms will not produce a regular diffraction pattern [63,69]. Instead, the diffraction pattern contains information from the contributions of a sample’s micro- and macro-structural features based on peak intensity data, information about the position and distance between atoms, temperature, space, and texture. It also can be used for quantitative phase analysis [69]. For example, PXRD analyzes the multi-component crystal formation mechanism and kinetics of a neutral diclofenac-L-proline multi-component crystal [70]. PXRD has become popular in solid pharmaceutical analysis due to the simplicity of sample preparation and the valuable data obtained regarding the crystalline drug phases [71].

Next, X-ray diffractometry is classified into PXRD (powder X-ray diffraction) and SCXRD (single crystal X-ray diffraction). PXRD differs from SCXRD in terms of sample preparation and instruments. PXRD measures the diffractogram of the powder sample, and SCXRD is used to determine a single crystal system’s new solid structure three-dimensionally. Hence, PXRD analysis is relatively faster than SCXRD, due to its more straightforward sample preparation steps. Practically, isolating single crystals with an appropriate size to be analyzed by SCXRD is challenging. However, PXRD requires a homogenous particle size with a dimension of less than ~10 μm. The small and homogenous crystal’s size can be randomly oriented, but the large variations in the particle size cause the broadening of the diffractogram and complicate structural assignments. In addition, PXRD requires an appropriate number of samples; meanwhile, SCXRD only needs one crystal but should be physically stable, with a size of about 0.1–0.5 mm [69]. SCXRD data collection is the final and crucial step and is used to reflect the reaction site and the non-covalent interactions, such as ionic or ionic neutral bonding.

SCXRD observes single or discrete diffraction peaks and transforms the peak positions into coordinates to recover the underlying crystal lattice dimensions or orientations commonly occurring in powder diffraction. As a result, the interpretation of single crystal diffraction is much less ambiguous than powder diffraction methods. Still, preparing the appropriate single crystal samples is challenging, laborious, and time-consuming. However, even though the crystal structure data are easier to interpret, the spatial properties of a single crystal may not represent the bulk solid, or appropriately describe the bulk properties of interest for a given application [72]. In this case, the structure’s image is commonly supported by software, such as SHELXL, SHELXT, and *Mercury*, in order to match the structure based on diffraction data, as measured by the goniometer [30,69,70]. 

Grinding with a mortar and pestle can be used for PXRD sample preparation, but it cannot be used for SCXRD, which requires a clear crystal. Therefore, only solvent-evaporation products are commonly used for SCXRD analysis. However, when using solvent evaporation, some hydrates and solvates may be formed [7,18,69]. Therefore, it is important to conduct an integrative study that combines various analytical methods to achieve the best results in the development of multi-component systems. This includes incorporating data from other solid analysis instruments in addition to PXRD measurements. PXRD measurements have been combined with other experiments under extreme conditions, such as high temperatures and pressures. This method may explore the material behavior changes as a function of these additional variables. For example, PXRD instruments have been integrated with DSC/DTA/TG, which is a widely used technique to investigate solid-state changes under different heating and pressure conditions, such as solvate/hydrate transformation, polymorphism, etc. [73]. PXRD can identify multi-component solids from their different diffractograms, rather than the component’s physical mixture. A diffractogram displays peaks at specific degrees of 2θ (x-axis) with varying intensities (y-axis) to determine crystallinity. Additionally, thermal analysis data, particularly TG, can help identify solvate and hydrate forms by calculating the mass decrease at the predicted water release temperature [18]. 

After a multi-component structure was obtained, some testing was conducted to evaluate the solid characters and properties, such as solubility, stability, hygroscopicity, etc. Moreover, some pharmacological aspects were checked, including activity, potency, and toxicity, to ensure the drug’s safety and efficacy. 

### 3.6. List of Antibiotic Multi-Component

Antibiotics have been reacted using various counterions/coformers to produce multi-component solid systems and have become the most developed compounds in multi-component solid form after NSAIDs. That fact is probably due to the high feasibility of their structure to react with other compounds. Secondly, there are several issues regarding the lack of physicochemical properties, such as solubility and stability, that need to be addressed. Fluoroquinolones are the second most studied group of antibiotics for multi-component solids after antituberculosis, as shown in Table 2. This table depicts some of the multi-component antibiotics, complete with their structure, stoichiometric proportion, preparation methods, benefits, activity change, and toxicity notes.

Table 2 shows some antibiotic multi-component crystals. For example, berberine HCl with fumaric acid [74] and nitrofurantoin–melamine [75] have increased stability. Next, researchers built many cocrystals of tuberculosis antibiotics to enhance their solubility and stability. Pyrazinamide and isoniazid are the specific antituberculosis agents developed in the multi-component solid by non-covalent reactions, such as that of the neutral combination of the antibiotic pyrazinamide and the preservative 3-hydroxybenzoic acid. The vibrational spectrometry data showed that the amino group of pyrazinamide forms a hydrogen bond at H_11_–N_12_–H_13_, with the -OH of the carboxylic acid of the conformer; this improved the physicochemical characteristics of the active pharmaceutical ingredient [68].

Additionally, from the cephalosporine group, cefixime was combined with nicotinamide to improve its solubility, dissolution, and stability [76]. Next, combining trimethoprim with 2,4-diaminopyrimidines pyrimethamine as a co-former built a crystal system with higher stability [80]. Afterward, sulfamethoxazole and succinimide multi-component solids showed higher stability than the base form [80,82]. In addition, cephalosporin with thymol produced a cocrystal that affected antibiotic activity [81]. 

Isoniazid has been combined with some acid substituents (p-aminobenzoic acid, p-cyanobenzoic acid, etc.) and plant metabolites (catechol, quercetin, etc.). This group of acid–coformer agents exhibited better solubility and stability and lower hepatotoxicity than the parent drug and the second group, which was a combination of isoniazid with some base substituents. Afterward, the combinations of prothionamide and phloroglucinol produced a new solid phase [78], which was also achieved with hydroquinone. Among the systems, prothionamide–adipic acid reached the highest concentration in 24 h [79]. 

The multi-component systems comprising isoniazid–acid substituents and natural resources were the following: isoniazid combined with cinnamic acid, fumaric acid [74,82,85,86], mandelic acid [82,91], glutaric acid [84], pimelic acid [84], malonic acid [84], hydroxybenzoic acid [86], adipic acid [84,86], gallic acid [86], gentisic acid [82,86], succinic acid and nicotinamide [88], glycolic acid [91], benzoic acid [90], ferulic acid [98], and oleanolic acid [93], etc., which some of them enhancing stability and solubility, and others decreasing those parameters. It has been hypothesized that the carboxylic acid substitution changes isoniazid’s stability and solubility. In addition, the formation of hydrates could decrease the solubility and stability of isoniazid and the natural substitutions. 

After the tuberculosis drugs, fluoroquinolones have become the most developed antibiotics, formed by non-covalent interactions with other components. Hereafter, the fluoroquinolone multi-component solid systems will be discussed in the following section, but we will still refer to the listing in Table 2. Moreover, a specific discussion about their combination with NSAIDs will be highlighted, including the possibility of altering their activity and the potential for drug–drug interactions and toxicity.

## 4. Fluoroquinolones Multi-Component Development

Fluoroquinolones are highlighted in this review because they are currently the most used antibiotics in common infectious bacterial diseases. Various fluoroquinolone derivatives show high effectiveness in killing bacteria. Structurally, their origin compound is nalidixic acid, with fluorine (F) added to the quinolone site, which produces a strong antibacterial effect. Ciprofloxacin, levofloxacin, ofloxacin, norfloxacin, lomefloxacin, moxifloxacin, pefloxacin, and sitafloxacin are the fluoroquinolones used clinically [4]. Those antibiotics are well absorbed and can be administered orally or parenterally [4]. However, this antibiotic group shows more effectiveness orally [23]. Fluoroquinolones act as bactericidal agents against a broad spectrum of bacteria, such as *Escherichia*, *Salmonella*, *Shigella*, *Enterobacter*, *Campylobacter*, *Neisseria*, and *Staphylococcus* [1]. In addition, fluoroquinolones kill *Neisseria gonorrhea* sp., which has been resistant to other antibiotics. Moreover, fluoroquinolones are also used to treat resistant *Salmonella* infections [36]. 

However, this antibiotic group generally has poor and pH-dependent solubility, besides exhibiting hydrate transformation, and is unstable in light [4,5]. Photo-degradation of these agents was reported to occur quickly, i.e., in one hour under UV lighting exposure. For example, levofloxacin was widely reported to degrade by UV radiation in both aqueous [103,104] and bulk solid form [105,106], as well as in a mixture with paracetamol [107]. The photolysis produced some toxic and allergenic products.

Another report explains that levofloxacin protected from daylight remained stable in 0.9% NaCl, 5% dextrose, and Ringer’s solution [5]. However, a slight decomposition of this antibiotic could still be observed after exposure to daylight, and Ringer’s solution was reported to show the fastest decomposition rate. The degradation product of levofloxacin was its N-oxide structure, from the oxidation of the methyl piperazine site, which is formed regardless of the solvent used. Hereafter, some efforts should be made to protect this compound from direct daylight [5,6,7,26,30]. 

Next, fluoroquinolone antibiotics are reported to have some hydrate forms, i.e., ciprofloxacin, levofloxacin, and sitafloxacin hydrates. The anhydrous levofloxacin absorbs some water molecules quickly and changes to a hemihydrate (levofloxacin 0.5 H_2_O) and then to a monohydrate (levofloxacin H_2_O). Inversely, in particular conditions, the hemihydrate could transform into anhydrous forms with various polymorphs (alpha, beta, gamma), or an amorphous form [19,58]. The different hydrate forms cause variability in physicochemical properties.

Fortunately, reports indicate that the instability towards light and the occurrence of pseudopolymorphism can be handled by reacting fluoroquinolone on the methyl–piperazine site with another component, with both ionic [6,7,30] and non-ionic interactions [26]. For example, a study combined levofloxacin with the antioxidant citric acid to form a salt, showing better photostability and increased antibiotic activity. In addition, citric acid protects levofloxacin’s N-methyl piperazine moiety from oxidative degradation [6]. Additionally, in other combinations, shown in Table 2, a quinolone derivative compound, berberine chloride, forms a multi-component salt with fumaric acid, which is more stable towards high temperatures and humidity than the starting drugs [74]. Next, the salt formation of levofloxacin with dihydroxybenzoic acids [7] and metacetamol 1:1 [26], as well as the combination of ciprofloxacin with a series of NSAIDs [30], also exhibits higher stability under humid and lit conditions than the fluoroquinolones [26]. 

Next, levofloxacin also has been combined with phthalimide and caffeic acid as a promising solid-state molecule, with higher antimicrobial efficiency and stability performance [53]. Other multi-component solids include the combination of ciprofloxacin with dicarboxylic acids, which improves the stability and solubility of the parent antibiotic. Additionally, the study also investigated the multi-component systems of ciprofloxacin with nicotinic acid and isonicotinic acid, in a stoichiometric ratio of 1:1, and the results were added to the data [61]. These systems were prepared using the grinding method and showed better solubility than the original compound [99]. In addition, the combined cocrystals of levofloxacin with stearic acid and sodium saccharin as co-formers also have been reported. In addition to the 1:1 molar ratio, a multi-component system with a 2:1 stoichiometric ratio of sodium levofloxacin–saccharin was also reported. This combination was found to increase the antimicrobial effect while maintaining stability. In addition, the 1:1 levofloxacin–stearic acid can increase the dissolution rate and mask the bitter taste [100].

Besides other organic compounds, the ionic reaction was conducted by metal substitution of the antibiotic levofloxacin. It was reported that the complexation of levofloxacin with copper (II) resulted in more stable levofloxacin. The antimicrobial potency test results showed the same activity as free-form levofloxacin [101]. However, it is necessary to continue testing levofloxacin against resistant microbes. Research on the effect of metal complexation of levofloxacin was also carried out on several transition metal ions, namely manganese, cobalt, nickel, copper, and zinc. It was reported that levofloxacin forms complexes with metal ions, forming bidentate ligands. Moreover, it was found that the antibacterial activity of these complex compounds was as good as the free form or better. In addition, those levofloxacin complexes increased the immune system’s activity (immunomodulator) [102].

Another combination system is the three-salt multi-component solid, with various hydrate molecules inserted. Ciprofloxacin (CfH, C_17_H_18_FN_3_O_3_) crystallizes with 2-thiobarbituric (H_2_tba) and barbituric acid (H_2_ba) in the aqueous solution to yield the salt CfH_2_(Htba)·3H_2_O, the salt cocrystal CfH_2_(Hba)(H_2_ba)·3H_2_O, and the salt CfH_2_(Hba)·H_2_O. These three salts were reported to have a more stable structure than the original compound [51]. 

Among the solid engineering techniques, solvate and hydrate developments are not good options, because the transformation causes a variation in the physicochemical properties, which is not easy to handle [56,57,58,59]. To enhance physicochemical properties, such as solubility and stability, tailormade salts and cocrystals are preferred [51,54,55]. Moreover, some reports revealed they might enhance antibiotic potency [30,52,53]. Hence, in the next subsequent section, we will explain the primary solid-state engineering of NSAIDs within a multi-component arrangement, including fluoroquinolones.

### 4.1. Fluoroquinolone-NSAID Multi-Components 

Like fluoroquinolones, NSAIDs have low solubility but high permeability and are classified in the Class II Biopharmaceutics Classification System (BCS) [8,21,29,30,70,108]. The poor solubility of NSAIDs impacts their’ performance, formulation, and clinical use, and prolongs the onset time. NSAIDs have been reported to form salts and cocrystals, as well as other multi-component systems such as polymorphs, solvates, and hydrates [8,54]. Due to their frequent usage, based on the data, NSAIDs are the most developed drug in solid-state engineering, followed by antibiotics, which have been designed to improve their solubility and stability [109].

Hereafter, next, we focus on fluoroquinolone–NSAID combinations for some reasons. First, it has been known that NSAIDs are essential substituents for clinical purposes, including in treating infectious diseases. Infected patients commonly use antibiotics to kill bacteria, and NSAIDs are used to overcome pain due to inflammation symptoms. Vice versa, inflammation also may trigger microbial infections. These drugs are available over the counter; therefore, NSAID usage is commonly not well-monitored, even though many adverse effects have been reported. Combining antibiotics with NSAIDs in a multi-component system will be better for increasing patient compliance [26,30]. 

The second supporting reason is structural; fluoroquinolones are amphoteric or tend to form bases [6,7], and are found in the market in their HCl–salt form. Meanwhile, NSAIDs are acidic or amphoteric, including diclofenac acid, which is commonly used in its sodium salt form [8,70]. However, combining them in a multi-component solid system is very challenging in improving the physicochemical properties of both sides, such as increased solubility and stability and the possibility of activity or potency enhancement. 

On the other hand, the salt reaction has limitations, because it cannot be applied to compounds that have difficulty forming ions. For example, an ibuprofen derivative, zaltoprofen, used to treat acute pains [110], is a non-ionic compound that cannot form salts. As an NSAID, this drug is poorly soluble and has been formulated as a sustained-release dosage form [111], mixed with a surfactant to increase the solubility [112]. However, this neutral compound can still be combined with nicotinamide via the non-ionic reaction to form a multi-component solid, which has been proven to improve its physicochemical properties [113]. Hence, there are still chances to react with weak base antibiotics. 

As mentioned earlier, NSAIDs and antibiotics are frequently used and have been found to react non-covalently with other compounds, which can offer both advantages and challenges [8,109]. A review of multi-component systems involving NSAIDs has been previously reported. On this occasion, we do not present the list of these agents but will continue discussing the possibility of their toxicity if combined with fluoroquinolones. 

Comparing drug–excipient combinations, not many multi-component dual drugs, including antibiotic–NSAID combinations, have been developed. This phenomenon is predicted, due to the complex factors affecting the feasibility of host–coformer interactions, safety, dose issue, and toxicity. Additionally, the data show that ciprofloxacin and levofloxacin are the most-developed fluoroquinolones, in the context of creating multi-component solids with NSAIDs. Ciprofloxacin is in the first rank, followed by levofloxacin because they are commonly used to treat broad infection cases and are easy to react with a counterion or coformer. Reports have widely explained that their main advantages comprise enhancing NSAID solubility and improving fluoroquinolone antibiotic stability [26,31]. 

For example, the ciprofloxacin-salicylic acid reaction produced various molar ratios of crystal, solvate, and hydrate forms of muli-component systems [31]. A study described these solid systems with a focus on the polymorphism, formation pathways, and thermodynamic stability of various other multi-component forms, including anhydrous salts, two polymorphic forms of monohydrate salt, methanol, and acetonitrile solvates, and hydrate cocrystal salts. Some of those multi-component systems provide better solubility and stability [31]. An experiment discovered ciprofloxacin–salicylate 1.75 hydrates, which enhanced the solubility of the original compound [65]. Other combinations include ciprofloxacin with mefenamic acid, tolfenamic acid, ketoprofen, dex-ketoprofen, and diclofenac acid, all of which improve the solubility and stability of NSAIDs. However, the ciprofloxacin–diclofenac salt exhibits the most potent antimicrobial effect [30]. 

In addition, as shown in Table 2, levofloxacin has been combined with metacetamol [26], citric acid [6], dihydroxybenzoic acid [7], and some inorganic metals, i.e., copper [101], argentum, etc. [102]. Besides, the HCl–salt forms have been used in their most marketed dosage forms. Similar to ciprofloxacin–NSAIDs, levofloxacin–NSAID multi-component solids also increase the anti-inflammatory drug’s solubility and stabilize the fluoroquinolone’s structure by protecting the N-methyl piperazine site, which is responsible for the photodegradation occurrence [26]. Besides the low solubility, instability towards light also becomes the main problem of fluoroquinolones [2,4,5]. Photolysis produces toxic substances immediately after UV exposure, in both liquid [103,104] and solid forms [105,106,107]. 

Briefly, the multi-component system of fluoroquinolones and NSAIDs has the potential to improve stability and maintain antibiotic potency. In the future, improved potency may also be achieved. However, as a dual drug, multi-component solid, interactions between APIs, mainly NSAIDs, should be considered thoroughly [114,115,116]. Many resistance cases related to the inappropriate use of levofloxacin have occurred widely [117]. The dose calculation and toxicity of the fluoroquinolone–NSAID combination, which has a role in its clinical potential, are crucial to be investigated [118]. 

### 4.2. Toxicity Study of NSAIDs and Fluoroquinolones Multi-Component Systems

Referring to other cases, apigenin, a bioflavonoid compound with anti-inflammatory activity against chronic aging diseases, has been combined with 4,4′-bipyridine, enhancing its efficacy and lowering toxicity [119]. In addition, the niflumic acid–pyridine multi-component solid successfully decreased gastrointestinal toxicity by resulting in the No Observed Adverse Effect Level (NOAEL) parameter [120]. However, many reports revealed cases of toxicity resulting from antibiotic–NSAID combinations, which depend on the dosage. High doses may significantly affect targeted cells, especially regarding the use of antibiotics and NSAIDs that have the same activity site, i.e., the neural system [118,121]. 

After discussing the antibiotic-NSAID multi-component solid, the following sections will focus on toxicity and computational studies. These studies aim to provide insights into selecting the appropriate host–coformer/counterion, preparation methods, structural determination, physicochemical properties, activity, and safety measures. Every drug has certain side effects, including antibiotics and NSAIDs, which may affect the gastrointestinal tract, nervous systems, and elimination organs, such as the liver and kidneys. For example, fluoroquinolones kill microbes by the inhibition of bacterial topoisomerases II and IV, and resistance arises from these target enzymes’ mutations. In addition, they have rare adverse effects, namely tendinopathy and tendon rupture, peripheral neuropathy, and aortic aneurysm [40]. Fluoroquinolones are selective GABA-A receptor inhibitors, preventing GABA binding in the central nervous system. As known, GABA is a primary regulator of the vagus nerve, which controls gastrointestinal (GI) function [3]. Moreover, fluoroquinolones also cause some neuro-system disturbances, such as seizures [121], hallucinations [122], peripheral neuropathy [123], and others [3].

On the other side, fluoroquinolones show many adverse effects on the gastrointestinal tract and the central nervous system, namely, phototoxicity and dermal toxicity, including allergenicity. Levofloxacin has favorable adverse reaction profiles compared to other fluoroquinolones. Among the reported cases of dermal toxicity, there are few reports of toxic epidermal necrosis (TEN) and stomatitis associated with levofloxacin usage. Stomatitis is characterized by pain, inflammation, and ulceration in the oral cavity [124]. 

There are mild to moderate side effects of fluoroquinolones, as shown when treated with ciprofloxacin, levofloxacin, and sitafloxacin groups. However, reports also reveal the severe side effects of temafloxacin, trovafloxacin, grepafloxacin, and clinafloxacin. For example, temafloxacin causes immune hemolytic anemia, trovafloxacin causes hepatotoxicity, grepafloxacin causes cardiotoxicity, and clinafloxacin causes phototoxicity. Therefore, those compounds are no longer used clinically. Additionally, regulatory agencies such as the FDA have warned about the inappropriate use of fluoroquinolone antibiotics, due to their severe side effects, such as tendon rupture, neuropathy, or heart valve regurgitation. Although the molecular principles underlying these side effects are not yet fully understood, there is evidence that they inhibit human mitochondrial topoisomerase II [3]. 

Another problem in antibiotic usage is bacterial resistance, which is divided into three categories, namely, multi-drug-resistant (MDR), extensively drug-resistant (XDR), and pan-drug-resistant (PDR) microbes. These categories illustrate the level of global complexity in the issue of antibiotic resistance and the many factors needed to be considered in solving it. Gram-negative bacteria are primarily XDR, followed by PDR and MDR. Recently, a study found resistant *Mycobacterium tuberculose*, including pre-XDR tuberculosis (TB) and MDR TB, which resist fluoroquinolones and are classically considered risk factors for treatment failure [125]. In these studies, combinations of plant extracts or active compounds have been reported to decrease antibiotic resistance issues. New research has shown that the treatment of extensively drug-resistant tuberculosis (XDR-TB) has been updated to include pre-XDR-TB, which is characterized by multidrug resistance (MDR-TB) with additional resistance to fluoroquinolone antibiotics. This was associated with more capillary lung lesions and bilateral disease, which required more prolonged treatment [125]. Also, more than half of *S. aureus* strains tested were MDR, showing resistance to fluoroquinolones [126,127,128]. Efforts have been made to improve the fluoroquinolones’ potency, for example, by conjugate biofilm formation, a major virulence factor of *Pseudomonas auroginosa* that causes antibiotic resistance [129].

On the other hand, side effects and toxicity of NSAIDs have also been reported. Most commonly, severe GI adverse effects increase with NSAID medication, such as ulceration, bleeding, or perforation [130]. The occurrences are more frequent in the elderly. These drugs also exhibit other undesirable effects, including nausea, dyspepsia, loss of appetite, abdominal pain, and diarrhea [131]. Furthermore, COX-1 inhibition reduces cytoprotective effects and causes these disturbances. In addition, NSAID usage may result in severe cardiovascular adverse events, i.e., myocardial infarction and stroke, particularly with the selective COX-2 inhibitor rofecoxib, which was withdrawn from circulation in 2004. These drugs have been found to elevate blood pressure and heart failure by inhibiting natural prostanoid-induced salt excretion and causing changes in renal arteriolar tone. Additionally, renal toxicity, including renal papillary necrosis and interstitial nephritis, also may be exhibited [132]. 

Aside from the singular NSAIDs, there are concurrent adverse effects related to other drug usage forms. Regarding their pharmacokinetics, NSAIDs may interact with other plasma protein-bound drugs and increase the free serum concentration of these medicines. In addition, NSAIDs decrease renal perfusion and inhibit the glucuronidation process. Therefore, their usage may increase the toxicity of drugs, in terms of renal clearance (such as lithium) or hepatic metabolism [116]. Furthermore, the concurrent use of NSAIDs and antihypertensives, anticoagulants, antiplatelets, selective serotonin receptor inhibitors (SSRIs), and substances that injure the GI mucosa also causes bleeding and severe ulcers due to decreased platelet aggregation. In addition, NSAIDs also interact with alcohol or glucocorticoids, which inhibits the activation of the arachidonic acid precursor phospholipase A2 and increases the risks of adverse effects [115]. 

The combination of fluoroquinolone with NSAIDs has been reported to increase the risk of seizure disorders, particularly in the central nervous system [32]. However, the mechanisms of toxicity in multi-component systems are still unclear and require further investigation. Due to differences in bonding and interaction, the proposed multi-component system has a unique working mechanism that can potentially impact toxicity. However, there are cases of decreases in toxicity after multi-component system formation. For example, a recent study reported that fluorouracil–phenylalanine cocrystal has different cytotoxicity from their single forms and physical mixture. The cytotoxicity mechanism was investigated in a cell metabolomics study, although it is still unclear [133]. The phytotoxicity of antibiotics and non-steroidal anti-inflammatory drugs to green algae *Chlorella* sp. and *Desmodesmus spinosus* also was reported [134]. Another study reported that oxaliplatin, an anticancer-multi-component system with the flavonoids baicalein and naringenin, could reduce toxicity [119]. The nano-cocrystal (NCC) technology of lamivudine and zidovudine was reported to have higher cytotoxicity. It was incorporated with a gel base delivery system, in NCC form [135].

Interestingly, a study reported that NSAIDs might increase the antibiotic’s strength, as shown by azithromycin–paracetamol co-crystal [136]. Next, the antibacterial and antibiofilm activities of four non-steroidal anti-inflammatory drugs (NSAIDs), piroxicam (PXC), diclofenac sodium (DCF), acetylsalicylic acid (ASA), and naproxen sodium (NPX), were evaluated against *Escherichia coli* and *Staphylococcus aureus*. Additionally, NSAIDs were combined with kanamycin (KAN) and tetracycline (TET). ASA, DCF, and PXC reduced metabolic activity and culturability. Meanwhile, PXC reduced biofilm mass. Additive interactions were obtained for most of the combinations between NSAIDs and KAN or TET. Hence, A benefit of NSAIDs is the control of biofilms, as they are more effective than conventional antibiotics [137]. In addition, recently, NSAIDs–ciprofloxacin salts, supported by computational and experimental studies, showed that NSAIDs not only maintain ciprofloxacin’s antibiotic activity but improve it by increasing the solubility, which then enhances their diffusion in the culture. This study shows that the reaction with diclofenac lowers ciprofloxacin’s required dose [30], as demonstrated by ciprofloxacin–furosemide combinations [138].

The exploration and investigation of toxicity and activity improvement by combining antibiotics and NSAIDs in a multi-component system is still a challenging task. However, the interactions between the two must be given attention, as they have shown similar adverse effects on the gastrointestinal tract based on available data. Therefore, for the next experiment, observing the possible pharmacokinetics and pharmacodynamic interactions and their impact on the neural, bloodstream, hepatic, and renal systems will be valuable for ensuring the safety and suitability of their multi-component solids. Furthermore, improvements in antibiotic activity and potency can be expected to be related to solubility and penetrability increases. 

As well as other synthetic drugs’ development, the relationship between structure and activity has been modeled in many ways, including by studying the activity and toxicity of antibiotic–NSAID multi-component solid systems, which should be confirmed by in vitro and in vivo testing. 

## 5. Computational Approach and Modeling for Multi-Component Solid Development

Computational simulations have become a popular and valuable technique for designing stable chain conformations and packing structures, predicting molecular interaction, etc. The computational approach in developing multi-component systems involves principles and specific calculations of the binding energy between multiple substituents. Molecular dynamics, mechanism, and quantum mechanics are some principles of multi-component solids’ design [29,33,39]. 

In line with experimental research, multi-component crystal building needs a suitable coformer, solvent, and preparation methods. Using a database, i.e., CSD (Cambridge’s Structural Database)–CCDC (Cambridge’s Crystallographic Data Centre), and mathematical calculation, the thermodynamic pattern of the multi-component system’s interaction can be predicted [139]. Based on these data, a coformer or counterion is selected. Next, computational modeling is used to determine feasible targets for multi-component crystals, predict the physicochemical properties of these systems, and optimize the preparation process. 

We have selected several multi-component systems as examples that have been modeled computationally and can be used as a basis for the development of antibiotics-NSAIDs multi-component systems. It is important to note that using in silico modeling is a more environmentally friendly and efficient approach compared to traditional trial-and-error experiments. Firstly, some programs have been used for solvent and coformer screening, such as *Mercury*, *Hyperchem*, COSMO-RS, COSMOtherm software, etc. [140].

For example, in the HME process for ciprofloxacin–isonicotinic acid multi-component development, a design of experiment (DoE) was used to evaluate the factors that determined the yield, and researchers found three factors: the effect of temperature, the screw speed, and screw configuration [99]. Furthermore, by analyzing the simulated structure, the activity change and toxicity can also be investigated simultaneously. 

A feasibility study was conducted using thermodynamic modeling, regarding the non-covalent reaction process, using perturbed-chain statistical associating fluid theory (PC-SAFT). This method can predict pharmaceutical cocrystal behavior by calculating the single-crystal solubility of any solvent at any temperature. Based on that calculation, the cocrystal’s solubility in other solvents and different temperatures also can be predicted without additional measurements. Using this mathematical approach, the properties of (+)-mandelic acid/(-)-mandelic acid (1:1), caffeine/glutaric acid (1:1), and carbamazepine/nicotinamide (1:1) cocrystal systems were in excellent agreement with the experimental data [140]. 

Developed in the early 1990s, the liquid phase thermodynamics theory of conductor-like screening model for real solvents (COSMO-RS) has been used in solubility prediction, solvent screening, excipient ranking, pKa prediction, redox potentials calculation, and partitioning coefficients’ determination [141]. In detail, the coformer screening is also based on some parameters: hydrogen bonding propensity, syntonic engineering, and supramolecular compatibility, which are supported by the Cambridge Structure Database (CSD), pKa-based models, Fabian’s method, lattice energy calculation, COSMO-RS, Hansen’s solubility parameter, virtual cocrystal screening (based upon molecular electrostatic potential surfaces (MEPS)), thermal analysis, measuring saturation temperature, Koffler contact method data, and so on [142].

Afterward, COSMO-RS was coupled with the COSMOtherm software to screen common solvents and co-formers for CL-20. The excess enthalpy and solvent cavity volume determined the solvate system’s arrangement. However, the COSMO method was challenging in predicting cocrystal formation, and it still needs entropy and solid-state interactions as the essential data. Meanwhile, solvent selection should maximize entropy. Calculating entropy is a critical factor in predicting cocrystal formation, and an accurate method is essential for screening cocrystal formation. This enables the design of an intelligent approach to multi-component crystal development [143]. 

Additionally, the supercooling liquid phase can be determined by in silico calculation [30]. A recent integrative study combined ciprofloxacin multi-component solids with a series of NSAIDs, including mefenamic acid, tolfenamic acid, dex-ketoprofen, ketoprofen, diclofenac, and sulindac, which successfully improved the NSAIDs’ solubility, without altering the antibiotic activity of ciprofloxacin. However, ciprofloxacin–DIC enhanced the antimicrobial activity. In the research on ciprofloxacin-NSAIDs multi-component solids, a virtual screening using COSMOQuick was conducted, which relied on thermodynamic calculations to select suitable NSAIDs to be combined with the antibiotic. This was achieved by determining the excess enthalpy of mixing or formation (Hex) of the components in comparison to the single drug in a supercooled liquid phase. A negative Hex value indicated the potential to form multi-component solids [30]. This computational program has also been used to search for new molecular complexes of furosemide–NSAIDs [138]. 

Next, an antibiotic, linezolid (LIN), was reported to produce many crystals named LIN_II, LIN_III, LIN:BA cocrystal, LIN:PHBA cocrystal hydrate, and LIN:2,6-DHBA cocrystal. Those crystal systems were obtained from some methods, including neat grinding, liquid-assisted grinding, and solvent evaporation, which fits with the in silico study. The virtual cocrystal-screening tools optimized the molecular complementarity, hydrogen bond propensity, and molecular electrostatic potential maps. However, the molecular electrostatic potential maps approach is closer to the experimental results than molecular complementarity and hydrogen bond propensity calculations. This work shows that the contribution of the total energy of the coformer crystal systems determines the cocrystal formation feasibility. Mercury 4.3.1 software provides data to analyze molecular complementarity [141]. 

Another modeling application in multi-component research is the study of spatial charge descriptors, which can predict cocrystal formation using machine learning algorithms [144]. Two models were developed to predict the density of energetic and general organic cocrystals containing nitro groups, based on the artificial neural network (model I) and surface electrostatic potential correction method (model II), used to predict cocrystal density. This study yielded reliable data, but the first model’s performance is better than model II’s [145]. 

Modeling software for designing multi-component systems is continuing to grow in number. Recently, Mswahili et al. developed a model to predict cocrystal formation by extracting the descriptor values from the simplified molecular-input line-entry system (SMILES) of compounds. They demonstrated that the calculated values of half of the selected descriptors using feature selection algorithms showed comparable results with experimental data from 1476 instances [35]. Furthermore, a prediction machine has been developed to focus on the L-menthol/thymol eutectic system, combining the data of differential scanning calorimetry (DSC) and powder X-ray diffraction (XRD). However, it was found that not all deep eutectic systems were simple eutectic types. Therefore, integrative experimental methods and thermodynamic modeling are required to determine the simple eutectic mixture [36]. 

Furthermore, new software prototypes have been developed to arrange multi-component systems by considering both thermodynamic and spatial aspects to fit the host compounds with their counterions/coformers. For example, a multi-component crystal may involve non-covalent bonds with low energy only, including hydrogen, van der Waals, and dipole–dipole bonds, until an ionic reaction, which forms relatively higher-energy salts Still, the space conformation and the environment also should be considered [30,99,143].

Modeling studies have played a significant role in the development of antibiotic multi-component crystals [30,37,99]. Adding to the mentioned studies, the stability modeling of sulfadimidine–4-aminosalicylic acid (4-ASA) cocrystals in two polymorphic forms and crystal habits has also been reported [37]. Next, in an antibiotic study, the use of an in silico method was applied to a multi-component system, 1-{(aryl) [(5-methyl-1,3-thiazol-2-aryl) amino] methyl naphthalene-2-ol, in order to promote some improvement in solubility and stability [38]. 

Another multi-component computational study was performed, combining antibiotic fluoroquinolone with metal by a dynamic and porin-mimetic approach. The metal–antibiotic system was predicted to improve solubility, which was then confirmed by the wet experiment [29]. Furthermore, fluoroquinolone complexes with Ag/AgCl/Bi_2_O_3_/BiFeO_3_, supported by modeling, showed photocatalytic degradation [102]. In silico approaches have been used to predict the formation of neutral multi-component crystals, such as the combination of quinolone antibiotics and a protein transporter (MexB) [146]. Similar approaches have been applied to other drug multi-component systems, including the prediction of cytotoxicity in fluorouracil–phenylalanine [133] and lamivudine–zidovudine [135] cocrystals. Another modeling study also discovered a new method for producing 5-amino-6-cyano-3-hydroxybenzoic coumarin, which shows antimicrobial activity, using Ni–Cu–Al–CO_3_ catalyst [147]. 

Finally, in the present, graphing a molecular structure three-dimensionally is usually assisted by SHELXT, and is refined using SHELXL software, combined with Mercury’s data and modeling [148,149]. All computational modeling approaches can be utilized to screen the coformers or counterions [150], design the preparation method [151], predict the physicochemical properties [152], and observe activity changes, even in the established structure [153]. Computational studies are essential to efficiently, rationally, and integrative develop suitable multi-component systems. In conclusion, in silico studies may produce a more accurate, greener, and comprehensive analysis, as shown by fluoroquinolones’ multi-component systems, regarding their reactions with NSAIDs [26,30,153], metals [29], and furosemide [138]. This modeling approach simplifies the conventional experimental process of selecting the coformer and method, determining the structure, and testing properties, resulting in a more efficient approach [154,155]. Moreover, it also can predict changes in potency, as widely reported in the current decades. 

## 6. Conclusions 

Besides resistance cases, antibiotics’ main problem is their lack of physicochemical properties, such as low solubility and stability. Therefore, one of the fruitful approaches in antibiotic development is solid-state engineering, i.e., the formation of multi-component systems composed to improve the stability and solubility of antibiotics. This strategy is growing in popularity and is supported by solid instrument development, straightforward preparation methods, and computational modeling, making it more efficient and environmentally friendlier. 

The most developed antibiotics in the multi-component solids are the antituberculosis group (isoniazid and pyrazinamide) and fluoroquinolones (levofloxacin and ciprofloxacin). Multi-component solid systems have several synthons that interact structurally to improve solubility and stability. However, based on the reports, many antituberculosis multi-component systems only result in new crystal structures without affecting stability and solubility. Meanwhile, fluoroquinolones react with their counterion or coformer at their amine piperazine moiety, which protects them from oxidative degradation. Moreover, fluoroquinolones’ multi-component solid systems may also increase their potency by re-arranging the polarity, supporting its capability to pass through the cell membrane of bacteria after the solid rearrangement. 

In conclusion, the development of the fluoroquinolone-NSAID multi-component solid system holds great promise and presents a challenging opportunity. This combination has the potential to leverage the advantages of both components and may lead to increased patient compliance. In general, multi-component solids overcome the low solubility and stability issues of NSAIDs. Moreover, some experiments focusing on improving the stability and polarity of these components may push antibiotic potency polarity. However, we also must pay attention to their safety factors by studying the toxicity, which may be assessed simultaneously by computational and laboratory studies.

## Figures and Tables

**Table 1:** Antibiotics groups summary [1,41,43,44,48,49].

No.	Antibiotics Group	Indication	Pharmacokinetic	Stability	Reference
1.	Sulfonamides (sulfisoxazole, sulfadiazine, sulfasalazine, sulfacetamide, mafenide, silver sulfadiazine, sulfadoxine)	Urinary tract infections, respiratory tract infections, gastrointestinal infections, pneumonia	70% to 100% of an oral dose is absorbed		[48]
2.	Trimethoprim	Urinary tract infections, respiratory tract infections, gastrointestinal infections, pneumonia	Distributed and concentrated rapidly in tissues, about 40% is bound to plasma protein.		[48]
3.	Sulfamethoxazole	Urinary tract infections, respiratory tract infections, gastrointestinal infections, pneumonia	About 65% is bound to plasma protein.		[48]
4.	Quinolone (nalidixic acid, ciprofloxacin, norfloxacin, levofloxacin, moxifloxacin, gatifloxacin, ofloxacin, pefloxacin)	Urinary tract infections, respiratory tract infections, gastrointestinal and abdominal infections, pneumonia, against anaerobic bacteria	They are well absorbed after oral administration and are distributed widely in body tissues.	Photosensitivity, hygroscopicity.	[48]
5.	Penicillins (penicillin G, penicillin V, ampicillin, amoxicillin)	Pneumococcal infections, Streptococcal infections, upper respiratory infections, urinary tract infections, and meningitis against anaerobic bacteria	Absorbed rapidly		[41]
6.	Cephalosporine (cefazolin, cefadroxil, cefoxitin, cefotetan, cefmetazole, ceftazidime, cefoperazone, cefepime)	Penicillin-resistant skin and soft tissue infections, respiratory tract infections, meningitis	Absorbed readily		[41]
7.	Aminoglycosides (gentamicin, tobramycin, amikacin, netilmicin, kanamycin, streptomycin, neomycin)	Bacterial endocarditis, tularemia, plague, tuberculosis, urinary tract infections, meningitis, peritoneal dialysis-associated peritonitis, sepsis	Very poorly absorbed from the gastrointestinal tract.		[44]
8.	Macrolides (erythromycin, clarithromycin, azithromycin)	Pneumonia, gonorrhea, pharyngitis, skin and skin-structure infections, sexually transmitted disease, diphtheria, pertussis	Erythromycin is absorbed adequately from the upper small intestine, and clarithromycin and azithromycin are absorbed rapidly from the gastrointestinal tract		[43]
9.	Clindamycin	Anaerobic bacterial infections, encephalitis	Nearly wholly absorbed following oral administration.		[43]
10.	Quinupristin/Dalfopristin	Vancomycin-resistant infections, complicated skin and skin structure infections, nosocomial pneumonia, methicillin-resistant infections	Administered by intravenous infusion		[43]
11.	Linezolid	Vancomycin-resistant infections, nosocomial pneumonia, community-acquired pneumonia, skin and skin structure infections	Well absorbed after oral administration, oral bioavailability approaching 100%		[43]
12.	Vancomycin	Pneumonia, empyema, endocarditis, osteomyelitis, soft-tissue abscesses, severe staphylococcal infections in patients who are allergic to penicillins and cephalosporins	It is poorly absorbed after oral administration, administered intravenously.		[49]
13.	Teicoplanin	Osteomyelitis, endocarditis	Administered intramuscularly, highly bound by plasma proteins (90–95%)		[1]
14.	Daptomycin	Complicated skin and skin structure infections, endocarditis, complicated bacteremia	It is poorly absorbed orally and administered intravenously.		[1]
15.	Bacitracin	Furunculosis, pyoderma, carbuncle, impetigo, superficial and deep abscesses, eczema, infected dermal ulcers	Administered topically		[1]
16.	Polymyxin	Multiple drug-resistant organisms infections, skin infections, external otitis, corneal ulcers infections	Administered topically, not absorbed when given orally, poorly absorbed from mucous membranes		[1]
17.	Mupirocin	Traumatic skin lesions, impetigo secondary infections, nosocomial infections, skin or soft tissue infections	minimal systemic absorption through intact skin or skin lesion; any absorbed drug is rapidly metabolized to inactive monic acid		[1]

**Table 2:** Antibiotics Multi-component Solids.

No.	Antibiotic Multi-Component	Structure	Preparation Method	Advantages	Other Information	Reference
1.	Berberine chloride–fumaric acid 1:1	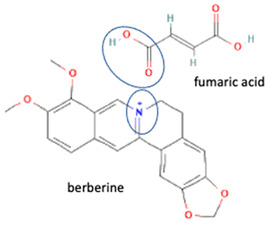	SL and SE at 20 °C	Better taste for an oral dose, increased stability towards temperature and humidity.		[74]
2.	Nitrofurantoin-melamine 1:1	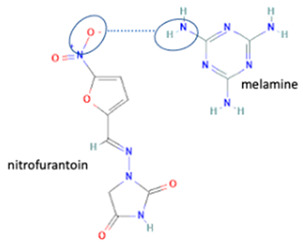	SE at room temperature	Increased nitrofurantoin’s stability		[75]
3.	Cefixime–nicotinamide 1:1	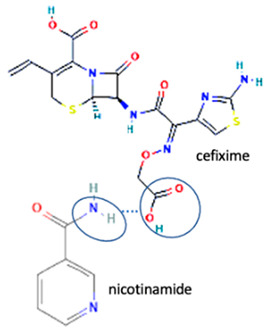	LAG	Improved solubility, dissolution, and permeability.		[76]
4.	Pyrazinamide–hydroxybenzoic acid 1:3	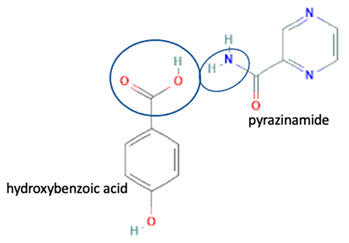	SE at room temperature using 20 mL methanol	Increased stability		[68]
5.	Pyrazinamide–1,4-dibromotetrafluorobenzene 2:1	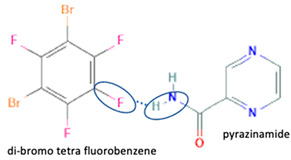	NG	New structure catalog		[77]
6.	Prothionamide–phloroglucinol 1:1	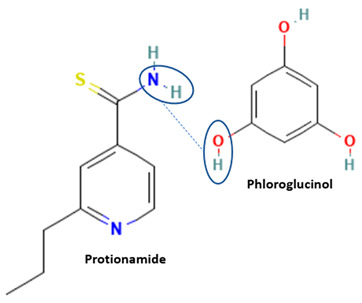	SE	Improved solubility		[78]
7.	Prothionamide–hydroquinone 1:1	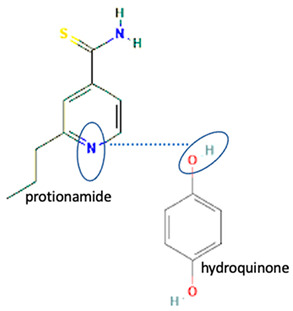	SE	Improved solubility		[79]
8.	Prothionamide–acid substituents (adipic acid, oxalic acetate, fumaric acid)	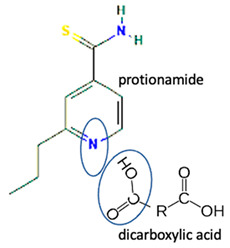	SE	Improved solubility		[79]
9.	Sulfamethoxazole–succinimide 1:1	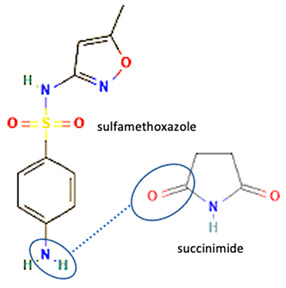	LAG using acetonitrile and SE at room temperature using ethyl acetate	Increased stability		[60]
10.	Trimethoprim-2,4 diaminopyrimidines	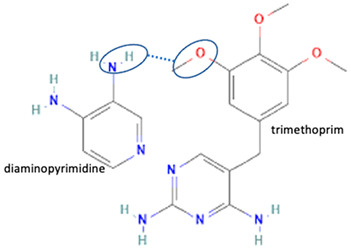	SE at room temperature	Increased stability		[80]
11.	Cephalosporin–thymol	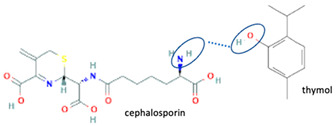	SE under a fume hood at room temperature			[81]
12.	Isoniazid–acid substituents (2,3-dihydroxybenzoic acid, 2,4-dihydroxycinnamic acid, 2,4-dihydroxybenzoic acid, 2-chloro-4-nitro benzoic acid, hydroxycinnamic acid) = 1:1	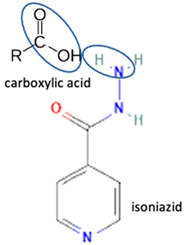	SE, LAG	New structure catalog	Antimicrobial effect decreases	[82]
13.	Isoniazid-2-methyl resorcinol-water 1:1:1 (And isoniazid with catechol, orcinol, pyrogallol, and phloroglucinol).	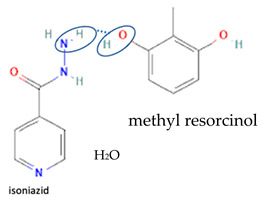	LAG with dichloromethane	New structure catalog	A cocrystal hydrate	[83]
14.	Isoniazid–catechol 1:1	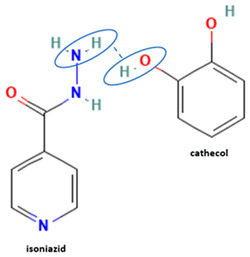	LAG with dichloromethane	Enhanced stability		[83]
15.	Isoniazid–orcinol 1:1	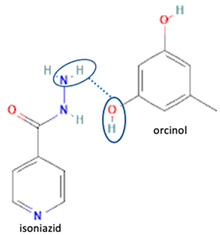	LAG with dichloromethane	New structure catalog		[83]
16.	Isoniazid–3-hydroxycinnamic acid 1:1	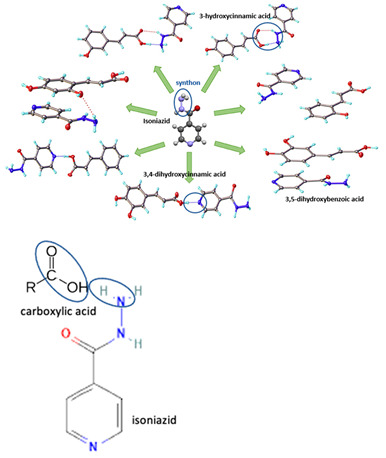	SE in methanol	New structure catalog		[82]
17.	Isoniazid–3,4-dihydroxycinnamic acid 1:1		LAG with methanol	The solubility of neutral multi-component systems tends to increase with increasing solubility of carboxylic acid structure.	Three forms: LAG with acetonitrile and further grinding gave form II. Form III was obtained by xylene/methanol SE at room temp.	[82]
18.	Isoniazid–3,5-dihydroxybenzoic acid-water 1:1:1		SE at room temp. In 2:1 ethanol: acetonitrile	New structure catalog		[84]
19.	Isoniazid–3-hydroxybenzoic acid–water 1:1:1	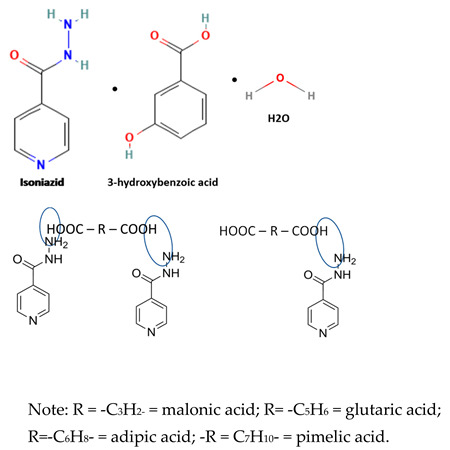	SE at room temp. In 2:1 ethanol: acetonitrile	New structure catalog		[84]
20.	Isoniazid–adipic acid 2:1		SE at room temp. Using methanol	Enhanced stability		[84]
21.	Isoniazid–glutaric acid 1:1		SE at room temp. Using methanol	New structure catalog		[84]
22.	Isoniazid–malonic acid 2:1		SE at room temp. Using methanol	Lower solubility and dissolution rate		[84]
23.	Isoniazid–pimelic acid 1:1		SE at room temp. Using methanol	Lower solubility compared to isoniazid; higher stability than isoniazid; structure.		[84]
24.	Isoniazid–4-hydroxycinnamic acid 1:1	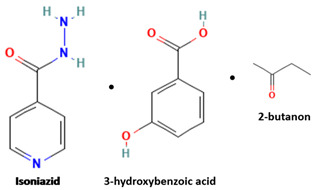	SE in methanol	The solubility of neutral multi-component systems tends to increase with the increasing solubility of carboxylic acid.		[85]
25.	Isoniazid–3-hydroxybenzoic acid- 2-butanone 1:1:1		SE at room temp. Using 2-butanone	New structure catalog		[86]
26.	Isoniazid–3-hydroxybenzoic acid- acetone 1:1:1	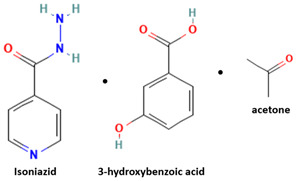	SE at room temp. Using acetone	Enhanced solubility and aqueous stability		[86]
27.	Isoniazid–4-aminosalicylic acid 1:1	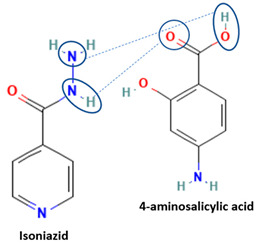	NG; LAG; SE using methanol	New structure catalog.	The hydrates have lower solubility and dissolution rates.	[87]
28.	Isoniazid–fumaric acid-pyrazinamide 1:1:1	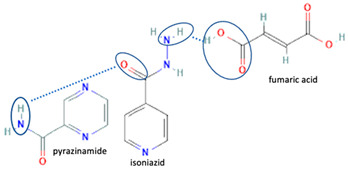	SE	Antioxidant, possibly shows an enhancement of stability for FDC	Drug–bridge–drug/ternary cocrystal	[88]
29.	Isoniazid–nicotinamide- fumaric acid 1:1:1	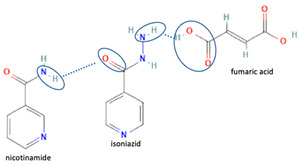	SE at room temp. Using methanol	Possible hepatoprotective effect	A ternary cocrystal	[88]
30.	Isoniazid–nicotinamide-succinic acid 1:1:1	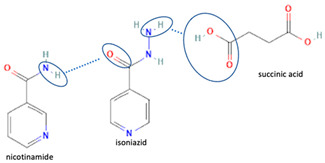	SE at room temp. Using methanol	New structure catalog	A ternary cocrystal	[88]
31.	Isoniazid–sebacic acid 2:1	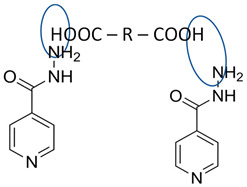 isoniazid—dicarboxylic acid (i.e., fumaric acid, sebacic acid, suberic acid)	SE in ethanol/acetonitrile (2:1 mixture)	Slower drug release; hepatoprotective effect		[89]
32.	Isoniazid–suberic acid 2:1		SE in ethanol/acetonitrile (2:1 mixture)	Enhanced stability		[89]
33.	Isoniazid–suberic acid 1:1		SE in acetonitrile/methyl tert-butyl ether	Enhanced stability		[89]
34.	Isoniazid–4-hydroxybenzoic acid-water (1:1:1 & 1:1:2)	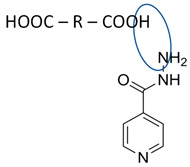 isoniazid with monocarboxylic acid (gallic acid, benzoic acid, cinnamic acid, ferulic acid, gentisic acid, glycolic acid, protocatechuic acid, mandelic acid, oleanolic acid).	SE at room temp.	New structure catalog		[84]
35.	Isoniazid–gallic acid 1:1		SE in 2:1 ethanol: acetonitrile; at room temp.	New structure catalog		[86]
36.	Isoniazid–benzoic acid 1:1		SE in ethanol/acetonitrile (2:1 mixture); ball milling	New structure catalog		[89,90]
37.	Isoniazid–cinnamic acid 2:1		SE in ethanol/acetonitrile (2:1 mixture) or LAG	Lower solubility and dissolution rate	Two polymorphs	[89]
38.	Isoniazid–ferulic acid 1:1		LAG in acetonitrile, further grinding resulted in Form 2, or by heating Form I to 130 °C for 30 min	Enhanced formulation and in-vitro/in-vivo synergistic effects	Two polymorphs	[86]
39.	Isoniazid–gentisic acid 1:1		SE in methanol	New structure catalog		[86]
40.	Isoniazid–glycolic acid 1:1		LAG	New structure catalog	A salt cocrystal	[91]
41.	Isoniazid–protocatechuic acid (3,4-dihydroxybenzoic acid)–water 1:1:1		SE at room temp. In methanol and water (1:1)	Slower drug release; hepatoprotective effect; enhanced bioavailability of quercetin	A hydrate	[92]
42.	Isoniazid–mandelic acid 1:1		LAG	Similar solubility		[86,91]
43.	Isoniazid–oleanolic acid 1:1		SE, LAG, and NG	New structure catalog		[93]
44.	Isoniazid–fumaric acid 1:1		SE at room temp. Using methanol	New structure catalog	Two forms /polymorphs	[88]
45.	Isoniazid–phloroglucinol 1:1	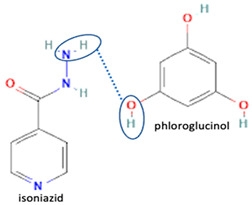	LAG	New structure catalog		[83]
46.	Isoniazid–pyrogallol 1:1	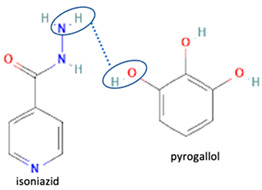	LAG with dichloromethane	Enhanced isoniazid stability		[83]
47.	Isoniazid–quercetin 1:1	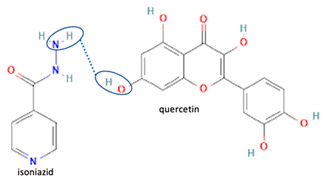	Anti-solvent using 2-propanol/n-hexane; LAG, followed by SE.		Lower solubility, local effect on the skin, the neutral multi-component system reduced the amount of permeated drug.	[94]
48.	Isoniazid–resorcinol	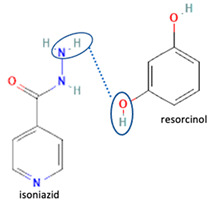	LAG using acetonitrile	The solubility of neutral multi-component systems tends to increase with increasing solubility of carboxylic acid; structure.		[94]
49.	Isoniazid–resveratrol 1:1	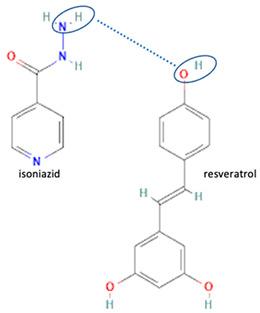	Reaction and crystallization using methanol	New structure catalog	Reactant products from isoniazid–resveratrol	[95]
50.	Isoniazid–p-aminobenzoic acid 1:2	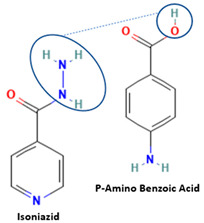	Two forms: Form I at 25 °C & II at −5 °CSE in 2:1 ethanol: acetonitrile	New structure catalog		[96]
51.	Isoniazid–p-cyanobenzoic acid 1:1	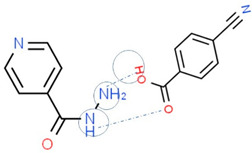	SE in 2:1 ethanol: acetonitrile	New structure catalog		[96]
52.	Isoniazid–p-nitrobenzoic acid 1:1	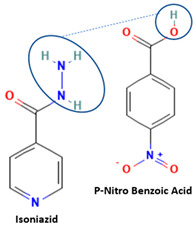	SE in ethanol	New structure catalog		[96]
53.	Isoniazid-oxalate 1:1	Isoniazid salts exhibit layered structures stabilized by N-H⋯O, C-H⋯O and π⋯π interactions. 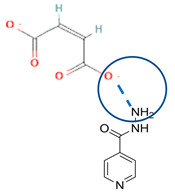 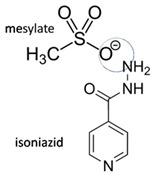	SE	Increases solubility and thermal stability		[97]
54.	Isoniazid-maleate 1:1		SE	Increases solubility		[97]
55.	Isoniazid–mesylate 1:1		SE	Increases solubility and thermal stability		[97]
56.	Isoniazid with–vanillic acid, ferulic acid, caffeic acid, and resorcinol	All cocrystal structures are sustained by the expected acid–pyridine synthon except the isostructural cocrystals, with the hydroxyl–pyridine synthon.	Grinding, slurry, heating	Increase solubility		[98]
57.	Ciprofloxacin, norfloxacin, and enrofloxacin with the α, ω-dicarboxylic acids glutaric acid, adipic acid, pimelic acid, suberic acid, azelaic acid, and sebacic acid.	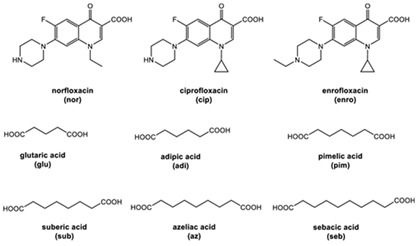 All salts and cocrystals contain the robust R_2_NH_2_^+^…–OOC or R_3_NH^+^…–OOC synthon. 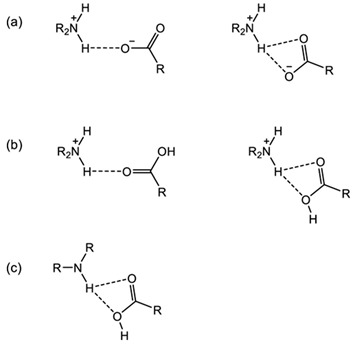 The reaction resulted in 27 new molecular salts and ternary molecular ionic cocrystals of compositions A^+^B^−^, A2^+^B^2−^, A2^+^B^2−^B, and A^+^B^−^A.	Solvent evaporation, LAG, and ball milling.	Increase solubility	Different stoichiomorphs, solvates, or polymorphs were obtained depending on the solvent. For example, the milled sample nor/az (1:1) was shown to gel the GRAS (generally recognized as safe) solvent propylene glycol, and enro/sub (1:1) was established into a gel xontaining both propylene glycol and water.	[9]
58.	Ciprofloxacin–tolfenamic acid 1:1	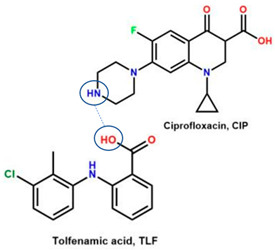	LAG	It enhanced the solubility of NSAIDs and ciprofloxacin’s stability.		[30]
59.	Ciprofloxacin–dexketoprofen 1:1	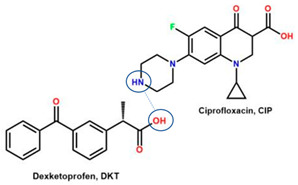	LAG	Enhanced the solubility of NSAIDs and ciprofloxacin’s stability.		[30]
60.	Ciprofloxacin–ketoprofen 1:1	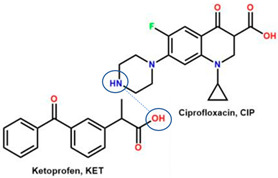	LAG	Enhanced the solubility of NSAIDs and ciprofloxacin’s stability.		[30]
61.	Ciprofloxacin–diclofenac 1:1	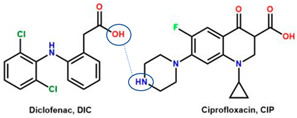	LAG	Enhanced the solubility of NSAIDs and ciprofloxacin’s stability; better efficiency (same antibiotic potency with reduced dosage)		[30]
62.	Ciprofloxacin–mefenamic acid 1:1	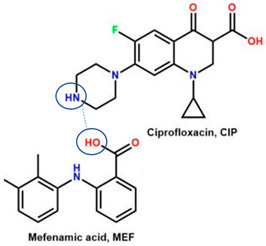	LAG	Enhanced solubility and thermal stability		[30]
63.	Ciprofloxacin–sulindac 1:1	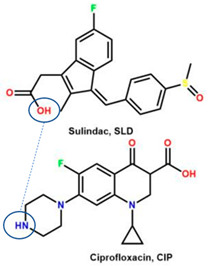	LAG	Enhanced solubility and thermal stability		[30]
64.	Ciprofloxacin–salicylic acid 1:1	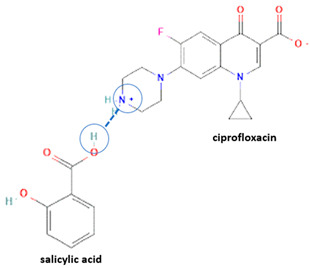	SE in a fume hood using methanol-water (1:1)	Increased solubility and dissolution		[31]
65.	Ciprofloxacin–salicylic acid–water (1:1:1)	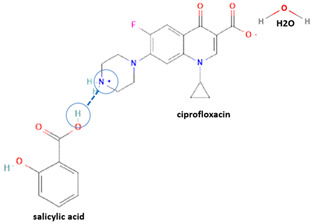	SE and FE	Increased stability and solubility		[31]
66.	Ciprofloxacin–salicylic acid–water (1:1:1.75)	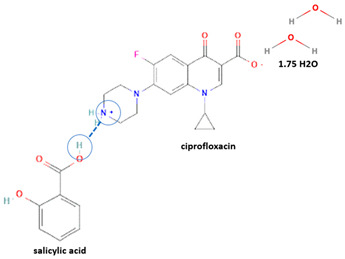	SE and FE	Increased stability towards humidity and solubility		[65]
67.	Ciprofloxacin–sodium-isonicotinic acid 1:1	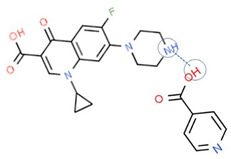	NG	Improved solubility		[61,99]
68.	Ciprofloxacin–acid substituents (dihydrobenzoic acid, 2-barbituric acid, barbituric acid, salicylic acid) 1:1	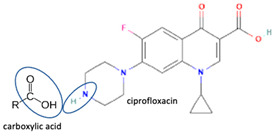	SE at room temperature	Increased solubility and stability	Adverse effect: The synergetic effect decreases as the dose increases	[11,51]
69.	Levofloxacin–metacetamol 1:1	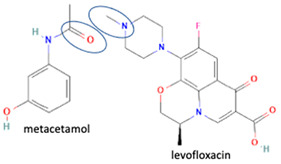	FE at 120 °C and SE at room temperature	Increased photostability against lighting and humidity		[26]
70.	Levofloxacin–citric acid 1:1 and 2,6-and 3,5-dihydroxybenzoic acid	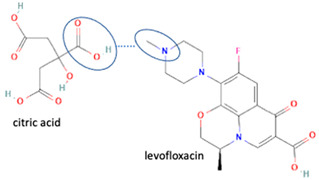	FE at 70–80 °C and SE at room temperature	Increased photostability and antibacterial effect		[6,7]
71.	Levofloxacin–sodium saccharin 1:1	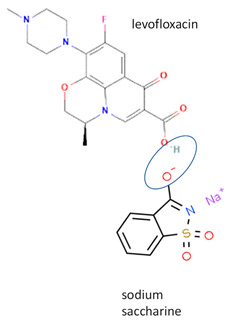	FE at 40 ± 2 °C in ethanol	Increased photostability and antibacterial effect	homosynthon interaction	[100]
72.	Levofloxacin–sodium saccharine 2:1	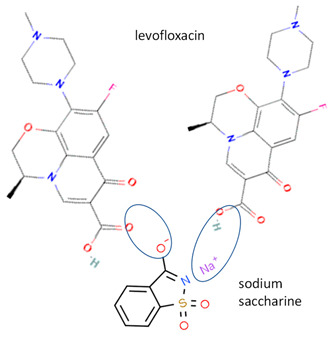	SE	Increased solubility		[100]
73.	Levofloxacin–stearic acid 1:1	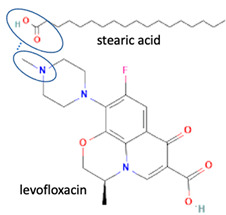	SE	Increased solubility		[100]
74.	Levofloxacin–metal copper 1:1	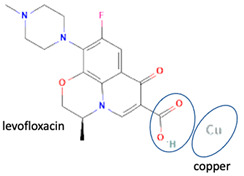 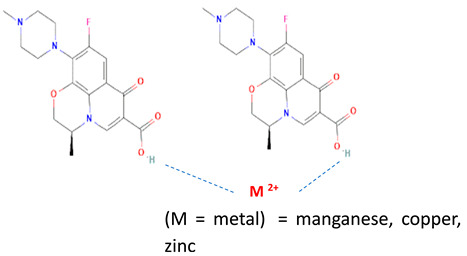	FE at 75 mBar and SE at room temperature using water-ethanol 1:1	Increased stability		[101]
75.	Levofloxacin with metal transition (manganese, cobalt, nickel, copper, and zinc) 2:1.		Increased stability and immunomodulator			[29]
76.	Levofloxacin–phthalimide–caffeic acid 1:1	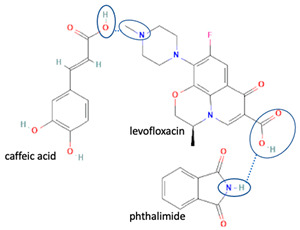	SE	Increased stability		[53]
77.	Levofloxacin hydrochloride -Ag^+^ ion 1:1	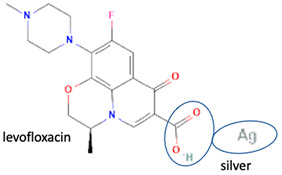	Complexation reaction	Increased photocatalysis	The antimicrobial effect was also increased	[102]

Note: SE = slow evaporation, FE = fast evaporation, LAG = liquid-assisted grinding, NG = neat grinding.

## Data Availability

Not applicable.
